# Sun Protective Behaviors and Attitudes of Runners

**DOI:** 10.3390/sports10010001

**Published:** 2021-12-21

**Authors:** Adam S. Tenforde, Michael Fredericson, Kierann E. S. Toth, Kristin L. Sainani

**Affiliations:** 1 Department of Physical Medicine and Rehabilitation, Spaulding Rehabilitation Hospital Harvard Medical School, Charlestown, MA 02129, USA; atenforde@mgh.harvard.edu; 2 Division of Physical Medicine and Rehabilitation, Department of Orthopaedic Surgery, Stanford University, Redwood City, CA 94063, USA; mfred2@stanford.edu; 3 Associates in Family Medicine, Fort Colins, CO 80524, USA; kierann.toth@gmail.com; 4 Division of Epidemiology, Department of Health Research and Policy, Stanford University, Redwood City, CA 94305, USA

**Keywords:** sunscreen, running, athletes, behaviors, photoprotection, skin cancer

## Abstract

Sun exposure is a risk factor for skin cancer. Knowledge and behaviors around sun exposure protective measures are poorly described in athletes including runners. Our primary objective was to describe sun exposure behaviors and knowledge in a population of runners. A cross-sectional online survey was administered to 697 runners to measure the frequency of seven sun protective behaviors: sunscreen use on the face or body; wearing a hat, sunglasses, or long sleeves; running in shade; and avoidance of midday running. Between 54% and 84% of runners reported that they engaged in these behaviors at least sometimes, but only 7% to 45% reported frequent use. Of 525 runners who gave a primary reason for not using sunscreen regularly, 49.0% cited forgetfulness; 17.3% cited discomfort; and only a small percentage cited maintaining a tan (6.1%) or optimizing vitamin D (5.1%). Of 689 runners who responded to a question about what factor most influences their overall sun exposure habits, 39.2% cited fear of skin cancer, 28.7% cited comfort level, and 15.8% cited fear of skin aging. In addition to the seven individual behaviors, we also asked runners how frequently they took precautions to protect against the sun overall. We explored associations between participant characteristics and the overall use of sun protection using ordinal logistic regression. Overall, sun protection was used more frequently in runners who were female, older, or had a history of skin cancer. Runners appear to recognize the importance of sun protection and the potential consequences of not using it, but report forgetfulness and discomfort as the biggest barriers to consistent use. Interventions using habit-formation strategies and self-regulation training may prove to be most useful in closing this gap between knowledge and practice.

## 1. Introduction

Skin cancer is a common form of malignancy in the United States. Outdoor athletes such as runners are at increased risk of skin cancer resulting from high sun exposure related to sports participation, especially during peak hours of ultraviolet (UV) radiation [1,2,3,4]. For example, National Collegiate Athlete Association (NCAA) division athletes may experience approximately 1000 h of sun exposure annually [5]. UV exposure results in a dose- and time-dependent induction of DNA damage [6], and sweating may intensify this effect [3]. Further, immunosuppression associated with overtraining syndrome in athletes has been proposed as a risk factor for the development of melanoma [7,8]. Compared with the general population, marathon runners present with more atypical nevi, especially those with highest training intensity [9]. These skin changes have been shown to be indicators of an increased risk for the development of melanoma [10,11].

Sun protective behaviors in the general population were reported in the U.S. National Health and Nutrition Examination Survey 2003–2006 [12]. In this study, 56% of Caucasians self-reported at least moderate (sometimes, most of the time, or always) use of sunscreen, while less than half of Hispanics and Blacks reported at least moderate use of sunscreen, hats, or long sleeves. We have limited knowledge of similar behaviors in outdoor athletes in the United States including runners. In a survey of collegiate athletes, 84% reported experiencing at least one sunburn in the past year and 43% reporting never using sunscreen [5]. Barriers to sunscreen use among collegiate athletes included not believing they were at increased risk of skin cancer, lack of concern about wrinkles or sunburns, desire to be tan, forgetting application, inconvenience of use, and expense. Studies in Europe inform the limited understanding of runner behavior. Behaviors of runners participating in endurance events in Porto, Portugal suggest a minority of runners surveyed had adequate sun exposure and women were more likely than men to engage in adequate behaviors [13]. Similar findings in a population of recreational runners in Switzerland who identified with a female gender, older age, and history of skin cancer were associated with greater UV protection [14]. A separate study in Spain suggested high prevalence of sun burns, desire for tanning, and limited use of protective clothing such as hats [15]. Behaviors and beliefs regarding sun protection in non-collegiate adult runners in the United States have not been well studied.

Our aim was to describe the sun protective habits in runners and determine the barriers to and motivations for using sun protection in a large adult population of runners in the United States. As a secondary analysis, we explored associations between participant characteristics and the overall use of sun protection.

## 2. Materials and Methods

We recruited subjects through the Pacific Association of USA Track and Field organization between January 2010 and March 2011. The association members are primarily from Northern California and part of Nevada. To be eligible, participants were 18 years of age or older and provided online consent prior to participation. The Stanford Institutional Review Board (protocol #17950) approved the research protocol.

The electronic online survey was designed to assess self-reported frequency and influence of behaviors related to sun protection, graded in categories with ability to select one response per question: never, seldom, sometimes, almost always, and always. Participants were asked about their use of 7 individual sun protective behaviors (wearing sunglasses, sun hat, or long sleeves; using sunscreen on the body or face; avoiding midday running; and running in the shade) as well as their use of sun protection overall (e.g., taking some precaution) when running outdoors during daylight hours. Additionally, subjects were asked to choose their primary reason for not using sunscreen if they do not use sunscreen regularly (from a menu of 7 choices), and they were asked to choose a primary influence on their sun exposure habits (from a menu of 5 choices). The questions are included in an Appendix A.

For descriptive purposes, we report frequencies and percentages in three categories: rare (never or seldom), sometimes, and frequent (almost always and always). We also created a composite variable of the 7 individual behaviors using principal components analysis. We calculated the Pearson’s correlation coefficient between this composite variable and the overall question to check for consistency. To explore the associations between participant characteristics and overall use of sun protection, we used ordinal logistic regression and included all 5 ordinal categories. We additionally used multiple linear regression to explore the associations between the participant characteristics and the composite of the 7 individual sun protective behaviors.

## 3. Results

### 3.1. Participants

In total, 697 runners (406 women and 291 men) participated in the study. At time of survey completion, runners were 42.3 ± 13.1 years old, ran 37.4 ± 17.7 miles per week in the past year, and had a body mass index of 21.9 ± 2.6 kg/m^2^. The full data set and statistical code is available as Supplementary File S1 and Supplementary Table S1.

### 3.2. Sun Protective Behaviors

The majority of runners reported using each of the seven assessed sun protective behaviors at least sometimes (Table 1); however, less than half reported frequent use (always or almost always) of each of the behaviors. Runners were most likely to frequently use sunglasses (45%), and least likely to frequently wear long sleeves (7%). Facial protection (sunglasses, facial sunscreen, hat) was more consistently used than full-body protection (sunscreen on the body, long sleeves).

A separate question characterized the primary reason for not using sunscreen while running. Of 525 runners who responded to this question, most cited forgetfulness (49%) or discomfort (17.3%), and only a small percentage cited maintaining a tan (6.1%) or optimizing vitamin D (5.1%) (Table 2). In total, 689 runners responded to a question about what factor most influences their overall sunscreen use and 39.2% cited fear of skin cancer, 28.7% cited comfort level, and 15.8% cited fear of skin aging (Table 3).

### 3.3. Factors Associated with Overall Use of Sun Protection

On the overall use of sun protection question, only 36% of runners reported frequently using sun protection, 35% reported sometimes using, and 29% reported rarely using. The composite measure of the seven individual behaviors using principal components analysis resulted in correlation between the overall sun protection question and this composite measure was 0.74.

We explored the association between participant characteristics and the frequency of use of sun protection. In univariable analyses, use of any sun protection was more frequent among females (*p* < 0.001), older runners (*p* = 0.008), those who ran fewer miles per week (*p* = 0.018), those with lower BMI (*p* = 0.007), and those who had a history of skin cancer (*p* < 0.001) (Table 4). However, mileage and BMI were no longer significant after adjusting for gender and age. A final multivariable model included: female gender (OR = 2.93, *p* < 0.001); older age (OR = 1.02 per year, *p* = 0.005); and a history of skin cancer (OR = 3.39, *p* < 0.001). In a multiple linear regression model that used the composite variable as the dependent variable, age, female gender, and history of skin cancer were also all robust predictors (*p* < 0.001 for all three variables).

## 4. Discussion

This study adds to limited knowledge on sun protective behaviors and attitudes among runners. A majority of runners used each of the seven sun protective behaviors at least sometimes. These rates are higher than have been reported in previous surveys of collegiate runners [5], other collegiate athletes [16], or in the general U.S. population [12]. Our population also appeared to be knowledgeable about the potential risks of sun exposure, identifying concerns for skin cancer or skin aging as the primary influence of their sun exposure behavior. However, a minority of runners reported frequent use of each of the seven sun protective behaviors or reported regular use of some form of sun protection. Thus, there appears to be a gap between knowledge and practice. Forgetfulness, discomfort, and inconvenience may be reasons for inconsistent use. Runners reported more consistent use for facial protection (facial sunscreen, hat, sunglasses) than for full-body protection (sunscreen on all exposed areas, wearing long sleeves).

Runners who were most likely to engage in sun protection included women, older runners, and those with history of skin cancer. The finding that women and older runners were more likely to use sun protection is consistent with prior reports [13,17,18]. It is also not surprising to identify that those with history of skin cancer reported greater sun protection behaviors. Similar to previous studies [5,13,18,19], we found less consistent sun protective use in males and younger runners, which suggests that intervention efforts that target these populations are needed.

Increases in knowledge about sun protection are not always tied to improvements in those behaviors [20]. Simple and effective methods that increase accessibility have been shown to increase sunscreen use, such as improved availability [16,21], text-message reminders [22], appearance-based interventions [23] (e.g., being shown a personal ultraviolet facial photograph) [24,25,26]. Our results similarly suggest that interventions that address issues of forgetfulness, comfort, and convenience may be effective. Further, the benefits of habit formation strategies and self-regulation training are a promising area of current research [27].

We note that the reported frequency of the overall use of sun protection is lower than the reported use of some specific individual behaviors (such as use of sunglasses). This may reflect differences in recall when asked to report an overall behavior versus individual behaviors. Runners may also engage in some protective behaviors for reasons other than sun protection; for example, they may wear sunglasses for visibility or appearance reasons. However, our sub-analysis does suggest good correspondence between the overall question and individual behaviors, suggesting internal validity of survey responses and that our survey is likely capturing the most common sun exposure protection strategies employed by runners.

While this is the largest study to understand health behaviors in recreational runners in the United States, we do recognize study limitations. We recruited from a running organization primarily based in Western United States with high sun exposure; thus, behaviors in our population may not be generalizable to runners in regions with different sun exposure. Further limitations may result from use of a convenience sample and self-reported behaviors. There is no established questionnaire on sun behaviors in runners and we did not validate our survey measures. To account for this, we measured individual responses to sun exposure behaviors to overall use of some form of sun protection and found strong agreement on principle component analysis. We collected responses in a finite time period and cannot verify whether more recent behaviors around sun protection have changed. Despite these limitations, our findings are novel in an understudied population and suggest runners may benefit from further efforts to reduce risks with sun exposure and develop further measures to evaluate changes in behavior that promote health.

## 5. Conclusions

Runners in this population fail to consistently use sun protection despite apparent recognition of the importance and potential consequences of not using it, reporting forgetfulness and discomfort as the biggest barriers to consistent use. Interventions utilizing habit-formation strategies and self-regulation training may prove to be most useful in closing this gap between knowledge and practice.

## Figures and Tables

**Table 1:** Runners’ frequency of use of sun protective behaviors while running. Frequent includes “always” or “almost always” and rare includes “never” or “seldom”.

Behavior	Frequent	Sometimes	Rare
Wear Sunglasses	45%	25%	30%
Avoid mid-day sun exposure (10 am to 2 pm)	43%	34%	22%
Wear Sunscreen on face	42%	25%	33%
Wear a hat which limits sun exposure	40%	28%	32%
Wear sunscreen on all exposed skin	18%	36%	46%
Run in the shade	10%	74%	16%
Wear long sleeves	7%	66%	28%

**Table 2:** Primary reason for not using sunscreen regularly (*n* = 525).

Forget	49.0%
Uncomfortable	17.3%
Avoid Mid-day sun	8.8%
Want to get tan	6.1%
Do not need or low risk	5.3%
Want to get more vitamin D	5.1%
Inconvenient or too lazy	4.0%
Other	4.4%

**Table 3:** Primary influence on sunscreen use (*n* = 689).

Fear of skin cancer	39.2%
Comfort level	28.7%
Fear of skin aging	15.8%
Optimize vitamin D	4.2%
Maintain tan	3.1%
Avoid sunburn	2.0%
Other	7.0%

**Table 4:** Associations of frequency of use of any sun protective behaviors with participant characteristics.

Characteristic	Frequent (*n* = 254)	Sometimes (*n* = 240)	Rare (*n* = 203)	*p*-Value
Gender				<0.001
Female	176 (69.3%)	149 (62.1%)	81 (39.9%)	
BMI	21.6 ± 2.5	22.0 ± 2.5	22.2 ± 3.0	0.007
Age	44.0 ± 12.2	41.7 ± 12.8	40.7 ± 14.4	0.008
Years of Education	15.9 ± 0.7	15.8 ± 0.8	15.8 ± 0.9	0.05
Ethnicity				
White	217 (85.8%)	208 (86.7%)	176 (86.7%)	0.16
Asian or Pacific Islander	24 (9.5%)	18 (7.5%)	12 (5.9%)	
Latino	5 (2.0%)	9 (3.8%)	10 (4.9%)	
Other	7 (2.8%)	5 (2.1%)	5 (2.5%)	
Miles Run Per Week (self-reported average)	36.1 ± 16.4	36.4 ± 17.5	40.1 ± 19.2	0.018
Highest Level of Competition Achieved in Running				0.14
Recreational	164 (64.6%)	140 (58.3%)	108 (53.2%)	
Conference/State	9 (3.5%)	15 (6.3%)	11 (5.4%)	
Regional	34 (13.4%)	39 (16.3%)	46 (22.7%)	
National	40 (15.8%)	33 (13.8%)	30 (14.8%)	
International	7 (2.8%)	13 (5.4%)	8 (3.9%)	
Stress (self-reported average; 1 = low, 10 = high)	5.9 ± 2.1	6.0 ± 2.1	5.6 ± 2.1	0.34
Sleep (self-reported average; hours per night)	7.3 ± 0.8	7.3 ± 0.9	7.2 ± 0.9	0.18
Previous Skin Cancer				<0.001
None	230 (90.6%)	231 (96.3%)	197 (97.0%)	
Any Skin Cancer	24 (9.5%)	9 (3.8%)	6 (3.0%)	

*p*-values are calculated using ordinal logistic regression preserving all 5 ordinal categories, but categories are collapsed into frequent (always or almost always), sometimes, or rare (seldom or never) for descriptive purposes.

## Data Availability

Deidentified data used in the study, as well as code for reproducing the statistics in the paper, are available in the supplement of this paper.

## Supplementary Materials

The following are available online at https://www.mdpi.com/article/10.3390/sports10010001/s1, File S1: Statistical code for analysis, Table S1: Deidentified data from participants.

## Appendix A. Questionnaire on Sun Exposure Behaviors

Answer questions for times when you are running outside during daylight hours.
How often do you wear sunscreen on your face? AlwaysAlmost alwaysSometimesSeldomNever
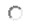

How often do you wear sunscreen on all exposed skin? AlwaysAlmost alwaysSometimesSeldomNever

How often do you wear a hat which limits sun exposure? Example: baseball hat, wide-brimmed hat? AlwaysAlmost alwaysSometimesSeldomNever

How often do you run in the shade? AlwaysAlmost alwaysSometimesSeldomNever

How often do you wear long sleeves? AlwaysAlmost alwaysSometimesSeldomNever

How often do you wear sunglasses? AlwaysAlmost alwaysSometimesSeldomNever

How often do you avoid mid-day sun exposure (10 am-2 pm)? AlwaysAlmost alwaysSometimesSeldomNever

How often do you take some precaution to protect your skin from sun exposure while running? AlwaysAlmost alwaysSometimesSeldomNever

If you DO NOT use sunscreen while running, what is the primary reason?	
	Cost
	Makes me sweat too much
	Runs in my eyes/mouth
	Want to get tan
	Want to get more Vitamin D
	Don’t think it prevents skin cancer
	Forget to put it on
	Other (Please Specify)
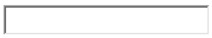

My sun exposure habits are primarily influenced by:	
	Fear of skin aging
	Fear of skin cancer
	Comfort level
	Optimize Vitamin D
	Maintaining my tan
	Other (Please Specify)
