# Supplementary material for: Sun Protective Behaviors and Attitudes of Runners
Source: Sports (Basel). 2021 Dec 21;10(1):1. doi: 10.3390/sports10010001 (PMC8822891; doi:10.3390/sports10010001)
Supplement: Supplementary file 1 [file sports-10-00001-s001.zip › Sun Protection in Runners Study SAS CODE(1).pdf]

File S1 Statistical code for analysis

```
/*Sun Protection in Runners Study*/
/*Data Analysis Updated October 2021*/
/*DataSet: sunprotection
Variables:
HighLevel: Highest Competitive Level
MPW: miles per week
Concrete: percent of time spent runnning on concrete
Hilly: percent of time running hills
Female: 1=female; 0=male
Ethn: Race/ethnicity
age: age in years
EduYrs: Years of education
Stress: Stress level on a 0-10 scale
Sleep: hours per night of sleep
BMI: body mass index
SkinCancer: 1=previous skin cancer (inc. melanoma), 0=no previous skin
cancer
SunscreenFace: sunscreen use on the face, 1=always; 2=almost always;
3=sometimes; 4=seldom; 5=never
SunscreenBody: sunscreen use on body, 1=always; 2=almost always;
3=sometimes; 4=seldom; 5=never
SunHat: wear a hat for sun protection, 1=always; 2=almost always;
3=sometimes; 4=seldom; 5=never
SunShade: run in shade for sun protection, 1=always; 2=almost always;
3=sometimes; 4=seldom; 5=never
SunLongSleeve: wear long sleeves for sun protection, 1=always;
2=almost always; 3=sometimes; 4=seldom; 5=never
SunGlasses: wear sunglasses for sun protection, 1=always; 2=almost
always; 3=sometimes; 4=seldom; 5=never
SunAvoidMidday: avoid midday sun, 1=always; 2=almost always;
3=sometimes; 4=seldom; 5=never
SunAnyProtection: overall use of sun protective measures, 1=always;
2=almost always; 3=sometimes; 4=seldom; 5=never
PrimaryReason: What is the primary reason that you don't use
sunscreen?
PrimaryInfluence: What is the primary influence on your sun exposure
habits?
*/

LIBNAME SUN BASE "C:\Users\kcobb\Desktop\sunscreen study" ;

/*Text of Results: Descriptive Statistics of the Sample*/
proc freq data=sun.sunprotection;
tables female;
run;
proc means data=sun.sunprotection n mean std;
var bmi age mpw;
run;

/*Table 1: Frequency of each type of sun protection*/
```

```
data temp;
set sun.sunprotection;
if sunscreenface=1 or sunscreenface=2 then sunscreenfacef="frequent";
if sunscreenface=3 then sunscreenfacef="sometimes";
if sunscreenface=4 or sunscreenface=5 then sunscreenfacef="rare";
if sunscreenbody=1 or sunscreenbody=2 then sunscreenbodyf="frequent";
if sunscreenbody=3 then sunscreenbodyf="sometimes";
if sunscreenbody=4 or sunscreenbody=5 then sunscreenbodyf="rare";
if sunhat=1 or sunhat=2 then sunhatf="frequent";
if sunhat=3 then sunhatf="sometimes";
if sunhat=4 or sunhat=5 then sunhatf="rare";
if sunshade=1 or sunshade=2 then sunshadef="frequent";
if sunshade=3 then sunshadef="sometimes";
if sunshade=4 or sunshade=5 then sunshadef="rare";
if sunlongsleeve=1 or sunlongsleeve=2 then sunlongsleevef="frequent";
if sunlongsleeve=3 then sunlongsleevef="sometimes";
if sunlongsleeve=4 or sunlongsleeve=5 then sunlongsleevef="rare";
if sunglasses=1 or sunglasses=2 then sunglassesf="frequent";
if sunglasses=3 then sunglassesf="sometimes";
if sunglasses=4 or sunglasses=5 then sunglassesf="rare";
if sunavoidmidday=1 or sunavoidmidday=2 then
sunavoidmiddayf="frequent";
if sunavoidmidday=3 then sunavoidmiddayf="sometimes";
if sunavoidmidday=4 or sunavoidmidday=5 then sunavoidmiddayf="rare";
if sunanyprotection=1 or sunanyprotection=2 then
sunanyprotectionf="frequent";
if sunanyprotection=3 then sunanyprotectionf="sometimes";
if sunanyprotection=4 or sunanyprotection=5 then
sunanyprotectionf="rare";
if highlevel="Recreational" then level=1;
if highlevel="Regional" then level=2;
if highlevel="Conference/State" then level=3;
if highlevel="National" then level=4;
if highlevel="International" then level=5;
run;
proc freq data=temp;
tables sunscreenfacef sunscreenbodyf sunhatf sunshadef sunlongsleevef
sunglassesf sunavoidmiddayf sunanyprotectionf;
run;

/*Table 2 and Table 3: Reasons for use or no use of sun protection*/
proc freq data=sun.sunprotection;
tables PrimaryReason PrimaryInfluence ;
run;

/*Text of Results: Calculate a composite measure of the 7 individual
behaviors using principal components analysis*/
proc factor method=principal rotate=varimax out=out factors=1;
var SunAvoidMidday SunGlasses SunHat SunLongSleeve sunscreenbody
sunscreenface sunshade;
run;
```

```
/*Text of Results: Calculate the correlation coefficient between the
overall question and this composite variable*/
proc corr data=out;
var SunAnyProtection factor1;
run;

/*Table 4: Characteristics by frequency of any sun protective
behavior*/
proc sort data=temp; by sunanyprotectionf;
proc means data=temp n mean std;
var age bmi eduyrs concrete mpw sleep stress ;
by sunanyprotectionf;
run;
proc freq data=temp;
tables female*sunanyprotectionf ethn*sunanyprotectionf
highlevel*sunanyprotectionf skincancer*SunAnyProtectionf
 /norow nopercent;
run;
%macro ord(var);
proc logistic data=temp;
model sunanyprotection = &var.;
run;
%mend;
%ord(age);
%ord(bmi);
%ord(eduyrs);
%ord(mpw);
%ord(sleep);
%ord(stress);
%ord(skincancer);
%ord(level);
%macro ord2(var);
proc logistic data=sun.sunprotection;
class &var.;
model sunanyprotection = &var.;
run;
%mend;
%ord2(ethn);
%ord2(skincancer);

/*Text of Results: Multivariable model/
/*Check whether BMI, MPW, and skincancer are predictors after
adjusting for age and gender*/
%macro ord3(var);
proc logistic data=sun.sunprotection;
model sunanyprotection = &var. age female;
run;
%mend;
%ord3(bmi);
%ord3(mpw);
%ord3(skincancer);
```

```
/*BMI and MPW are not significant after accounting for age and gender,
so these are not included in the final multivariable model*/
/*Final model includes age, female, and history of skin cancer*/
proc logistic data=sun.sunprotection;
model sunanyprotection = skincancer age female;
run;

/*Text of Results: Confirm these predictors using the composite
variable as the dependent variable in linear regression*/
proc reg data=out;
model factor1= skincancer age female;
run;
```
